# Revalidation of a modified and safe approach of stellate ganglion block

**DOI:** 10.4103/0019-5049.76601

**Published:** 2011

**Authors:** Ashok Jadon

**Affiliations:** Senior Consultant and HOD, Anaesthesia, Tata Motors Hospital, Jamshedpur, Jharkhand, India

**Keywords:** CRPS-I, modified technique, stellate ganglion block

## Abstract

Stellate ganglion block (SGB) is very effective in management of chronic regional pain syndrome (CRPS-1). However, serious complication may occur due to accidental intravascular (intra-arterial) injection of local anaesthetic agents. Abdi and others, has suggested a modified technique in which fluoroscopy-guided block is given at the junction of uncinate process and body of vertebra at C7 level. In this approach vascular structures remain away from the trajectory of needle and thus avoid accidental vascular injection. We have used this technique of SGB in nine patients who were treated for CRPS-I. The blocks were effective in all the patients all the time without any vascular or other serious complication.

## INTRODUCTION

Stellate ganglion block (SGB) is an effective technique to manage patients of chronic regional pain syndromes type 1 (CRPS-1).[1,2] Previously anterior paratracheal technique[3] was the standard technique to block stellate ganglion (SG). But this technique may result in number of serious, some time life-threatening complications for e.g., subarachnoid or epidural injection, recurrent laryngeal nerve block, seizures, blindness, locked-in syndrome.[4,5] Although various new techniques like computerized tomography, magnetic resonance imaging and radionuclide tracers have been suggested to facilitate SGB, it is difficult to use these techniques in routine clinical practice. Fluoroscopic-guided block which is easy to use and also provide safety is now the practical and appropriate choice for SGB.[6]

The SGB is most commonly done at C6 level as this level provides well defined landmarks and relative safety against inadvertent intravascular injection. However, blockade of SG at C6 may result in failure or unsuccessful block as there is always a possibility that sympathetic supply of upper limb may not be completely blocked. Studies have suggested that for complete block of sympathetic supply of upper limb it is necessary to give injection at C7. Moreover thermographical assessments of the sympathetic blockade by SG have shown that C7-SGB is more effective than C6-SGB.[6,7] But as the C7 vertebra has no anterior tubercle and palpation is difficult, injury to the pleura and to the vertebral artery is common especially with the blind technique. Therefore newer and safer techniques have been suggested.[8]

Abdi and others[8] described an oblique fluoroscopic approach targeting the junction between the uncinate process and the vertebral body at the C7 level to block SG effectively and avoiding vascular injury. To revalidate the safety and efficacy of this fluoroscopic-guided technique of SGB we used this technique in 19 blocks given in nine patients who were treated for upper limb CRPS-1.

## METHODS

Nine patients (male=2, female=7) of age 43.3±13.6 yrs (22-62yrs) were given SGB to manage symptoms of CRPS-1 [Table 1]. All the patients after confirmed diagnosis were initially managed by orthopaedic departments and received treatment with analgesics, anti-inflammatory medicine and physiotherapy for 2-4 months. After clinical physical examination and review of investigation informed consent was obtained. All the procedures were done in afternoon and patients were allowed to have breakfast in morning and water till 2 hrs before procedure.

**Table 1:** Patient’s age, sex, parts affected, duration of symptoms, number of blocks and complications during individual block

Case no. #	Age and sex	Area affected	Duration of symptoms	Associated cause of CRPS	No. of blocks	Complication on number of block
1.	54 yrs F	Right upper limb	4 months	Road traffic accident (RTA) #upper end humerus	3	Nil-1 and 3, Dysphasia-2
2.	22 yrs F	Left hand	2 months	RTA left hand	3	Hoarseness-1, Nil-2 and 3
3.	45 yrs F	Right hand	3 months	After close reduction for #collies	2	Nil-1 and 2
4.	62 yrs F	Right upper limb	3 months	Peri-arthritis shoulder	3	Nil-1 and 2, Giddiness-3
5.	45 yrs M	Left upper limb	3 months	RTA with crush injury hand	2	Hoarseness-1 Nil- 2
6.	28 yrs F	Right hand	4 months	Internal fixation #lower end radius	1	Dryness in throat-1
7.	37 yrs F	Left hand	3 months	Minor injury in hand due to fall	2	Nil-1 and 2
8.	35 yrs M	Right hand	3 months	RTA with crush injury	2	Dysphasia-1, Nil-2
9.	60 yrs F	Right hand	4 months	#Both bone, internal fixation	1	Dyspnoea-1

CRPS: Chronic regional pain syndromes; RTA: Road traffic accident;

# Fracture

### Technique

We used the similar technique as advised by Abdi and others.[8] Intravenous line with 20-G IV canulae was secured. SGB was performed with the patient in the supine position with the neck slightly extended (a pillow may be placed beneath the shoulders), and the head rotated slightly to the opposite side to be blocked [Figure 1a]. Patients were monitored (pulse-oximeter and blood pressure) by multipara monitor (Infinity Vista XL, USA). The skin temperatures were recorded in the distal portion of both the upper extremities in mirror-image locations [Table 2]. The fluoroscopy beam was directed in an anteroposterior direction with caudocra-nial angulations of the C-arm. The C-arm is then rotated obliquely, to the side where blockade is desired [Figure 1b]. The rotation must occur to allow adequate visualization of the neural foramina [Figure 1c]. A skin wheal was raised at the surface point where the junction of the uncinate process and the vertebral body is seen on the fluoroscope [Figure 1c]. Under real-time imaging, a single pass is made with a 25-G spinal needle to contact bone at this point. In its final position, the nee-dle tip comes to rest at the junction be-tween the uncinate process and the verte-bral body [Figure 2a]. The stylet is removed, the extension set is attached and 1-2 ml of radio-opaque contrast is injected to vi-sualize the longus colli muscle [Figure 2b]. After nega-tive aspiration is performed, a 0.5-mL test dose of 1% xylocaine is injected to rule out intravascu-lar injection into the vertebral artery and then mixture of 1% Xylocaine (10 ml)+ 40 mg Depomedrol ® was injected. Flow of contrast extending to the head of the first rib can be observed by increasing the amount of contrast or injection of drug mixture which pushes the contrast up and down [Figure 2c].

**Table 2:** Changes in temperature of affected and contralateral limb; before and after stellate ganglion block

Block number	Temperatures of affected limb °C			Temperatures of contralateral limb °C			Temperature difference °C between two limbs
	Before block	After block	Temperature difference	Before block	After block	Temperature difference	
1.	33.1	34.7	1.6	32.8	33.1	0.3	1.3
2.	33.4	34.9	1.5	33.8	33.9	0.1	1.4
3.	32.9	35.0	2.1	32.4	32.6	0.2	1.9
4.	32.4	34.1	1.7	32.4	32.5	0.1	1.6
5.	33.0	36.1	3.1	33.0	33.6	0.6	2.5
6.	33.1	35.4	2.3	32.9	32.9	0.0	2.3
7.	32.8	34.4	1.6	32.8	32.8	0.0	1.6
8.	33.3	34.8	1.5	33.3	33.5	0.2	1.3
9.	31.9	33.9	2.0	31.0	31.0	0.0	2.0
10.	32.4	34.1	1.7	33	33.4	0.4	1.3
11.	33.2	35.1	1.9	33.2	33.5	0.3	1.6
12.	30.0	31.2	1.2	30.0	30.0	0.0	1.2
13.	30.1	33.0	2.9	30.3	30.5	0.2	2.7
14.	31.3	34.3	3.0	31.0	31.4	0.4	2.6
15.	32.4	34.1	1.7	31.7	32.2	0.5	1.2
16.	30.6	31.8	1.2	30.0	30.2	0.2	1.0
17.	29.8	32.0	2.2	30.0	30.4	0.4	1.8
18.	31.3	32.9	1.6	30.9	31.0	0.1	1.5
19.	32.4	33.5	1.1	32.0	32.0	0.0	1.1

**Figure 1a:**
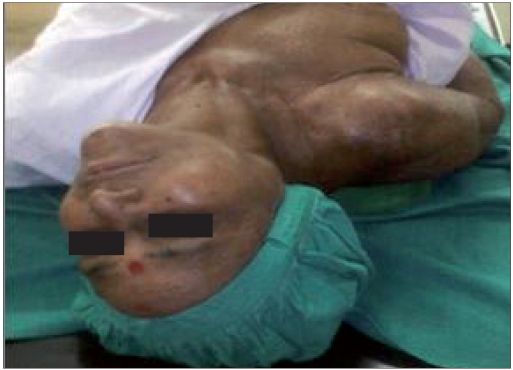
Neck extended and rotated to opposite side

**Figure 1b:**
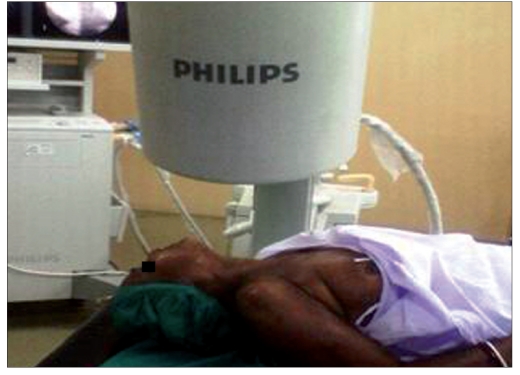
C-arm is rotated in opposite direction of head turn

**Figure 1c:**
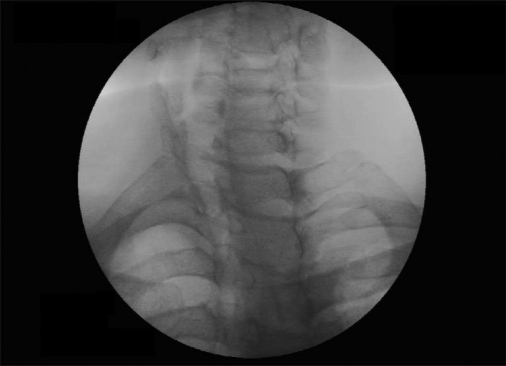
X-ray image shows uncinate process of C7 and intervertebral foramina

**Figure 2a:**
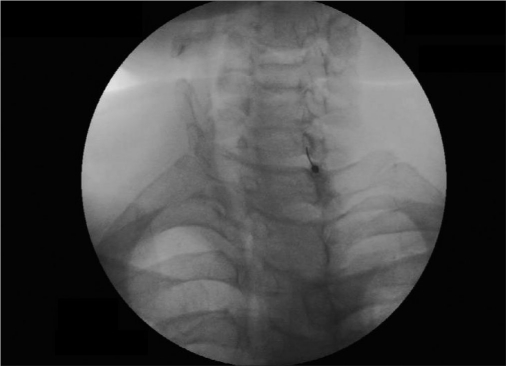
spinal needle at uncinate process of C7 left

**Figure 2b:**
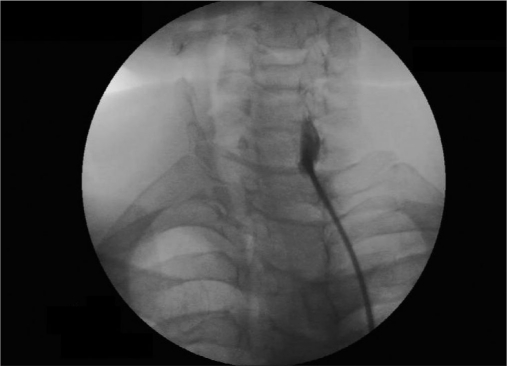
Contrast spreading up and down

**Figure 2c:**
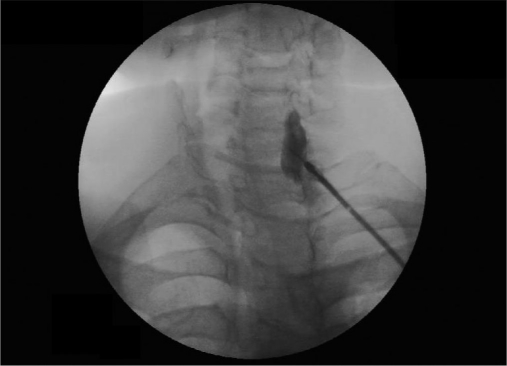
Contrast spreading up to head of 1^st^ rib

Patients were observed in recovery area for 1 hr and then discharged. Review was done once weekly to evaluate pain intensity (VAS 0-10) and range of movements (ROM).[9] Blocks were repeated 1-3 weekly interval depending upon relief of symptoms and patients willingness.

## RESULTS

Seven out of nine patients (2 males and 5 females) showed marked improvement in symptoms during follow-up. One young girl (case 2) although responded to treatment (objective improvement in colour and swelling) but did not get full ROM. She was seeking an outside referral and thus referred for corrective surgery of hand. Case 9 refused to treatment after first injection where she developed dyspnoea.

Few minor and short-lived side effects such as temporary hoarseness of voice, difficulty in swallowing and Horner’s syndrome were noticed [Table 1]. One patient (case 9) developed dyspnoea. There was no evident cause of dyspnoea except nasal congestion and a patient has to breathe through mouth. Vitals and SpO_2_remained normal. Pneumothorax was ruled out by chest auscultation and fluoroscopy. Patient responded to reassurance and oxygen by mask for 10 minutes. No patient had any vascular injury or developed any other serious complication during or after procedure.

## DISCUSSION

Although SGB is an important tool to manage sympathetically mediated pain in the upper extremity. There are two concerns regarding its success.

Firstly, to block the SG effectively for CRPS-1 of upper extremity it is essential to inject the drug near ganglion. There are a significant number of individuals in whom the intrathoracic somatic branches from the second thoracic spinal nerve join the first thoracic spinal nerve. These fibers (Kuntz’s nerves) join the lower part of the brachial plexus without passing through the SG. This explains the incomplete sympathetic blockade of the upper extremities in SGB.[10] This happens because if drug is injected higher up (at C6) it may not reach up to target area.[6] It has been shown in one study that methylene blue injection with the classical blind ap-proach at C6 was thoracoscopically visu-alized at the SG in only 46%, while with the same blind technique at C7 the dye was seen at the SG in 63% of cases.[11]

Secondly, there is always a possibility of serious consequences of intravascular (intra-arterial injection) due to anatomy at C7specialy when blind technique is used. Other fluoroscopic-guided techniques[12,13] also enhance safety. However, manual retraction of vascular sheath always poses practical difficulty of needle displacement while taking hands off for attaching the extension tube or syringe for contrast injection. This new modified technique has better (hands free) approach. The advantages of this new technique are as follows:[8]

- Eliminates pressing or pushing the vascular system out of the way
- Eliminates pressing on the Chassaignac tubercle, which can be uncomfortable and even painful for patients
- Minimizes the chance of intravascular injection
- Minimizes the chance of oesophageal perforation
- Reduces the volume of local anaesthetics needed to cover lower cervical through upper thoracic areas
- There are many case reports of intravascular injection and related complications with classical techniques.[14,15] However; actual incidence of intravascular injection during classical approach of SGB is not given in literature. The incidence of vascular injury during SGB between 0 and 30% has been reported by various authors.[16,17]

We noticed in our study that once C-arm is positioned correctly the localization of target point (the junction between uncinate process and body of vertebra) was achieved in all the patients in single attempt except to correct final angle in few patients.

## CONCLUSIONS

Our results of nine patients simply revalidate that this new technique of SGB is safe and effective technique of SGB to manage CRPS-1 of upper limb. However, studies with large number of patients and randomized control trials can only substantiate the claim of its superiority over other similar technique.
